# The Combined Use of Lenvatinib and Locoregional Therapies for the Management of Hepatocellular Carcinoma

**DOI:** 10.3390/cancers17091572

**Published:** 2025-05-05

**Authors:** Ronit Juthani, Pannaga Malalur, Ashish Manne, Arjun Mittra

**Affiliations:** 1 Department of Internal Medicine, Saint Vincent Hospital, Worcester, MA 01608, USA; ronit.juthani@stvincenthospital.com; 2 Department of Internal Medicine, Division of Medical Oncology, The Ohio State University Comprehensive Cancer Center, Columbus, OH 43210, USA; pannaga.malalur@osumc.edu (P.M.); ashish.manne@osumc.edu (A.M.)

**Keywords:** TACE, TARE, Y90, liver cancer, immunotherapy, TKI, embolization

## Abstract

Hepatocellular carcinoma (HCC) is a commonly diagnosed malignancy, with the treatment for transplant-ineligible localized disease traditionally relying on locoregional therapies, such as surgical resection, transarterial chemoembolization (TACE), and transarterial radioembolization (TARE). Systemic therapy has historically been reserved for advanced, unresectable HCC. However, lenvatinib, an oral multikinase inhibitor, has recently gained traction as part of a multimodal approach for localized HCC in combination with locoregional treatments. An upfront TACE or TARE can induce tumor hypoxia, leading to the upregulation of hypoxia-inducible factor-1 alpha (HIF-1α) and vascular endothelial growth factor (VEGF), which promotes tumor angiogenesis and progression. The rationale for combining lenvatinib with a locoregional therapy is to enhance tumor shrinkage while preserving liver function before a definitive intervention. Clinical trials, such as TACTICS and LAUNCH, have demonstrated improved outcomes with this approach. Additionally, retrospective studies, including those incorporating immune checkpoint inhibitors, have reported further benefits. This review explores the combination of lenvatinib with various locoregional modalities, including TARE, microwave ablation (MWA), and radiofrequency ablation (RFA), highlighting their indications and clinical outcomes. Furthermore, we discuss the ongoing and upcoming clinical trials investigating the integration of systemic agents with locoregional therapies for intermediate-stage HCC, including EMERALD-1, EMERALD-3, LEAP-012, and CheckMate 74W.

## Simple Summary

Hepatocellular carcinoma (HCC) is one of the most common and life-threatening cancers worldwide. Many patients cannot undergo surgery due to advanced cancer, requiring radiation, targeted therapy, or immunotherapy instead. Localized HCC can be treated by blocking the blood supply to the tumor using particles or beads containing chemotherapy or radiotherapy agents targeting the blood vessels supplying the tumor. In the last few years, researchers and clinicians have explored utilizing a combination of these localized agents in addition to a targeted drug called lenvatinib for localized HCC to improve outcomes. Clinical trials and retrospective studies have suggested that this combination approach allows for tumor shrinkage while preserving liver function, allowing for more effective treatment. Ongoing clinical trials are also evaluating the addition of agents that activate the immune system along with this combination; if successful, they could lead to better treatment options for patients with HCC.

## 1. Introduction

Hepatocellular carcinoma (HCC) represents the sixth most common cancer worldwide [1]. In terms of mortality, it ranks fourth only to lung, colorectal, and stomach cancers, with a very high mortality rate. HCC represents 75–85% of primary liver cancers worldwide [1], with more than 800,000 people diagnosed and more than 700,000 succumbing to the disease every year. According to the American Cancer Society, in 2025 in the United States (US), an estimated 42,240 new cases of liver and intrahepatic bile cancers will be diagnosed and 30,090 deaths are expected [2]. Liver cirrhosis is the primary risk factor for HCC, with one-third of cirrhotic patients expected to develop HCC during their lifetimes [3]. The annual incidence ranges from 2% in hepatitis-B-infected cirrhotic patients and 3–8% in hepatitis-C-infected cirrhotic patients to a lower incidence of 1.5% among patients with cirrhosis secondary to the metabolic dysfunction-associated fatty liver disease (MAFLD) spectrum [4]. The surveillance of high-risk patients using ultrasound and computed tomography scans along with serum alpha-fetoprotein (AFP) levels allows for the detection of HCC at an early stage when potentially curative options are available [4].

The treatment options for HCC have typically been guided by the use of the Barcelona Clinic Liver Cancer (BCLC) staging system, which takes into account the liver function, performance status, tumor characteristics (number and size of lesions), and the presence of metastasis to classify patients as very early stage (BCLC 0), early stage (BCLC A), intermediate stage (BCLC B), advanced stage (BCLC C), or terminal stage (BCLC D), with varying treatment options [5]. The management of transplant-ineligible patients without metastasis has traditionally been focused on locoregional modalities (LRMs), such as ablation, resection, transarterial chemoembolization (TACE), transarterial radioembolization (TARE), and stereotactic body radiation therapy (SBRT). Systemic therapy has usually been reserved for HCC that has progressed to an advanced stage with preserved liver function (based on the Child–Pugh score, which is used to assess the severity of liver disease in those with cirrhosis) [6]. In the last few years, the idea of using systemic therapy early, along with locoregional therapy, has gained traction. Specifically, the use of lenvatinib, an oral multikinase inhibitor that was earlier reserved for advanced HCC, is now being considered for localized HCC [7,8]. Combining systemic therapy with LRMs is fast gaining interest for HCC management. Here, we discuss the advances in localized HCC treatment over the last decade using lenvatinib and LRMs.

## 2. Lenvatinib

Lenvatinib is a multikinase inhibitor that inhibits vascular endothelial growth factors (VEGFs) 1, 2, and 3, as well as other tyrosine kinase receptors, such as fibroblast growth factor receptors (FGFRs) 1, 2, 3, and 4; platelet-derived growth factor receptor α (PDGFR α); cKIT; and RET; which are involved in promoting cancer via proangiogenic and oncogenic pathways [9]. In in vitro studies, the primary mechanism of action through which lenvatinib exerted its antitumor activity was by suppressing proliferation signals from the VEGF receptor (VEGFR) and FGFR pathways [10]. Apart from the differences in their binding to different kinase receptors, the major distinguishing feature that separates sorafenib and lenvatinib is the latter’s potent activity against FGFRs 1–4. Lenvatinib and sorafenib also regulate the expression of matrix metalloproteinase (MMP) and the tissue inhibitors of MMPs (TIMPs), thereby inhibiting the invasion and metastasis of human HCC cells [11]. Lenvatinib was first evaluated in a noninferiority trial with sorafenib (the REFLECT trial). Lenvatinib was found to be noninferior, with a significantly better progression-free survival (PFS) (7.4 vs. 3.7 months) in advanced HCC [12]. The median overall survival (OS) was comparable between the two groups (13.6 months in the lenvatinib group vs. 12.3 months in the sorafenib group). Based on the findings of the REFLECT trial, lenvatinib was approved by the US Food and Drug Administration (FDA) in 2018 as a first-line treatment for patients with advanced HCC [13]. The European Society of Medical Oncology (ESMO) guidelines recommend using lenvatinib as a first-line treatment option in patients with HCC without invasion of the main portal vein and who have an Eastern Cooperative Oncology Group (ECOG) performance status of 0–1 [14]. The National Comprehensive Cancer Network (NCCN) recommends using lenvatinib in patients with HCC and Child–Turcotte–Pugh-A (CTP-A); in those with unresectable disease who are not candidates for a transplant; and metastatic disease and an extensive tumor burden. The most common adverse event (AE) seen with lenvatinib was hypertension, predominantly occurring due to vascular endothelial dysfunction. The maximum tolerated dose of lenvatinib in the phase 1 clinical trial was determined to be 12 mg daily. In the REFLECT study, the following frequencies of AEs were noted in the lenvatinib group: hypertension (42%), diarrhea (39%), decreased appetite (34%), and weight loss (31%) [14].

Subsequently, with the development of immunotherapy combinations for HCC treatment, such as atezolizumab–bevacizumab, the OS and the objective response rate (ORR) of lenvatinib were less favorable than those of the combination immunotherapies [15]. Lenvatinib fell further down the pecking order when it was compared with the immune checkpoint combination of durvalumab plus tremelimumab in the HIMALAYA trial; the OS and ORR were significantly better for the new combination in this trial [16]. Contraindications to immunotherapy can preclude the use of these newer combination drugs, while lenvatinib and sorafenib maintain their appropriateness. There may also be a possible role for lenvatinib as a second-line treatment option in patients who show disease progress on atezolizumab–bevacizumab. Yoo et al. conducted a retrospective study of 49 patients with disease progression who were on this combination, and subsequently used, as a second-line treatment, lenvatinib, sorafenib, or cabozantinib; they found that the group that received lenvatinib exhibited a longer median PFS compared to those treated with sorafenib (6.1 vs. 2.5 months), although there was no marked difference in the OS (*p* = 0.347) [17]. This has also been incorporated into the latest NCCN, American Society of Clinical Oncology (ASCO), and ESMO guidelines. Lenvatinib has also been tested in clinical trials along with pembrolizumab based on the principle of its synergistic effect with pembrolizumab; by inhibiting angiogenesis and the immune-suppressive nature of the tumor microenvironment they thereby boosted the immune response [18]. In the phase 3 LEAP-002 trial, which evaluated the efficacy of lenvatinib plus pembrolizumab compared to lenvatinib alone in advanced HCC, the median OS and PFS for lenvatinib plus pembrolizumab were 21.1 months and 8.2 months, respectively, compared to 19 months and 8.1 months, respectively, for lenvatinib monotherapy. While this is not a significant difference, the subgroup analysis revealed that the patients most likely to benefit from the combination treatment were those with high-risk features, including macrovascular invasion/extrahepatic spread (hazard ratio (HR): 0.78) and an elevated AFP status (HR: 0.67) [19,20]. Recently, the role of lenvatinib has been explored in localized intrahepatic HCC in combination with locoregional therapies. This represents an exciting new avenue that is capable of revolutionizing HCC treatment.

## 3. Lenvatinib Combined with Locoregional Therapies

The addition of systemic therapies to locoregional therapies in intermediate-to-advanced HCC has yielded important results and improvement in the overall treatment efficacy. The data have been especially groundbreaking for the combinations involving lenvatinib and TACE. The fundamental principle of this combination stems from the fact that lenvatinib induces tumor shrinkage and the preservation of liver function before any definitive TACE can be undertaken. The upfront use of TACE in HCC with a high tumor burden may sometimes be incomplete, increasing tumor hypoxia and the upregulation of hypoxia-inducible factor-1α (HIF1α),VEGFs, and PDGFRs [21]. These factors increase tumor angiogenesis. Lenvatinib, in turn, inhibits VEGFRs 1, 2, and 3 and PDGFRs, and serves as a complement to TACE, reducing post-TACE VEGF and PDGF overexpression, thereby enhancing the effectiveness of TACE [21]. Using lenvatinib in such a situation normalizes the vasculature and allows for a uniform distribution of lipiodol-containing anticancer drugs within the tumor.

This section covers the various combinations of lenvatinib with different locoregional therapies and the outcomes of those studies.

### 3.1. Lenvatinib with TACE

#### 3.1.1. Retrospective Studies Exploring Lenvatinib and TACE

The first breakthrough study exploring the role of systemic therapy combined with TACE was the TACTICS trial, a randomized clinical trial (RCT) comparing TACE plus sorafenib versus TACE alone in a first-line setting for unresectable HCC [12]. It included patients with one or more measurable target liver lesions based on RECIST, classified as BCLC B or C and Child–Pugh-A. Patients were excluded if they had received prior systemic therapy or if they had greater than 50% liver function, bile duct invasion, or portal vein invasion at the main portal vein. TACE plus sorafenib had a significantly longer PFS than the TACE-alone group (25.2 vs. 13.5 months; *p* = 0.006). The OS at 1 year and 2 years in the TACE plus sorafenib compared to sorafenib alone was 96.2% and 82.7% vs. 77.2% and 64.6%, respectively. Following this, Fu et al. conducted a retrospective study comparing the clinical outcomes of patients receiving TACE plus lenvatinib vs. lenvatinib monotherapy for unresectable HCC with a Child–Pugh A or B classification; they observed a significant improvement in the TACE plus lenvatinib group in terms of the 1- and 2-year OS (1-year OS: 88.4% vs. 79.2%; 2-year OS: 79.8% vs. 49.2%; *p* = 0.047); PFS (1-year PFS: 78.4% vs. 64.7%; 2-year PFS: 45.5% vs. 38.0%; *p* < 0.001); and the ORR (ORR: 68.3% vs. 31.7%; *p* < 0.001) [22]. The best ORR was also significantly improved in the TACE plus lenvatinib group (68.3% vs. 31.7%; *p* < 0.001). The benefit of sequential therapy with TACE was also explored by Kawamura et al. in advanced HCC with Child–Pugh class A liver function and BCLC stage A-C tumors with a treatment interval of greater than 28 days since a prior TKI inhibitor therapy; they found from a subgroup analysis that the use of lenvatinib–TACE sequential treatment after progression during lenvatinib monotherapy was associated with better post-progression survival [23].

Another study conducted by Ando et al. evaluated the benefit of adding lenvatinib for 88 patients with intermediate-stage HCC, and found that after propensity score matching, while the ORR (63.2 vs. 63.2%) and median PFS (11.6 months vs. 10.1 months) were not significantly different in the lenvatinib–TACE group vs. the TACE group, the median OS was significantly higher in the lenvatinib–TACE group than the lenvatinib-alone group (not reached vs. 16.9 months; *p* = 0.007). Transaminitis and fever were more frequent in the lenvatinib–TACE group, although they did not result in significant morbidity and mortality [24]. Tada et al. studied early lenvatinib administration in 208 patients with intermediate-stage HCC using inverse probability weighting and found that early lenvatinib administration improved the 6-,12-, 18-, and 24-month cumulative survival rates significantly (*p* < 0.001). A univariate analysis also demonstrated that lenvatinib therapy was associated with a significantly increased OS in certain patients with HCC (HCC beyond the up-to-7 criteria—the sum of the size of the largest tumor (in centimeters) and the number of tumors exceeding 7) (HR: 0.230; *p* = 0.035) [25]. These findings were also reinforced by Shimose et al., who performed a retrospective propensity-matched analysis of 113 lenvatinib-treated patients with intermediate-stage HCC and found that the survival rates of the patients who received TACE were significantly higher than patients who did not receive it (median survival time: not reached vs. 16.3 months; *p* = 0.01). The one- and two-year survival rates after PSM were significantly higher in the group receiving TACE (83% and 66% vs. 71% and 28%, respectively). From a Cox regression analysis, the independent factors associated with the OS were receiving TACE and an albumin–bilirubin score grade of 1 [26]. Chen et al. performed a small but important retrospective study in 2022, reviewing 12 consecutive patients with HCC and portal vein tumor thrombosis (PVTT) along with Child–Pugh A or B liver function, and found that the median OS and PFS were 16.9 and 6.15 months—an improvement upon the findings from the REFLECT trial [27]. Another retrospective study of TACE combined with lenvatinib compared to TACE alone in BCLC B2-stage HCC was conducted by Liu et al. In their study of 66 patients, lenvatinib and TACE had a significantly longer OS compared to TACE alone (HR: 0.395; *p* = 0.023) [28]. The 2-year OS was also significantly higher in the lenvatinib plus TACE group (76.3% vs. 45.4%). The patients that benefitted the most according to the subgroup analysis were patients > 60 years old; those with lower levels of AFP (≤400 ng/mL); more than three tumors, with the largest tumor size < 4 cm; and those without hepatitis B infection and liver cirrhosis. There was no significant difference between the two groups in terms of the median PFS. The tumor response, however, was significantly better in the lenvatinib plus TACE group, with an ORR of 64.7% compared to 34.4% in the TACE-alone group (*p* = 0.014). Lin et al. added an interesting facet by examining the effect of locoregional therapy on the survival of patients with unresectable HCC on lenvatinib treatment [29]. They found that, compared to those with tumor progression without locoregional therapy, the patients who had received locoregional therapy after lenvatinib treatment experienced significantly better survival rates (cumulative survival rate: 70% vs. 27%; *p* = 0.003).

Wang et al. performed a retrospective analysis of 65 patients with unresectable HCC comparing the addition of pembrolizumab and lenvatinib along with TACE to lenvatinib and TACE alone [30]. This involved patients with BCLC A, B, or C HCC and Child–Pugh class A or B liver function. They found that receiving pembrolizumab with lenvatinib was associated with a longer OS compared to those who received lenvatinib alone (26.8 months vs. 14.0 months, *p* = 0.027). There was also a significant improvement in PFS in the combined lenvatinib-pembrolizumab group (11.7 months vs. 8.5 months, *p* = 0.028). However, no significant ORR difference was noted, although the disease control rate (DCR) improved significantly (93.3% vs. 64.0%, *p* = 0.003).

Comparisons have also been made regarding the use of two different tyrosine kinase inhibitors (TKIs) combined with TACE. Yang et al. performed a prospective cohort study analyzing the combination of TACE plus lenvatinib vs. TACE plus sorafenib for unresectable HCC with PVTT and liver function with Child–Pugh A or B. They found that while there was no significant difference in the OS and PFS rates between these two groups from the raw analysis, there was a significant improvement in the OS and PFS, after the propensity score-matching analysis, in the TACE–lenvatinib group (OS: 18.97 months vs. 10.77 months; HR—2.21, *p* = 0.022; PFS: 10.6 months vs. 5.4 months; HR—2.62, *p* = 0.003). The ORR was also significantly better in the TACE–lenvatinib group after the propensity-matched analysis (66.8 vs. 33.3%, *p* = 0.037) [31].

The addition of newer therapies to the combination of lenvatinib and TACE has also been tested in recent years. Tang et al. recently published an article studying the combination of TACE with lenvatinib and the PD-1 inhibitor camrelizumab vs. TACE alone for its efficacy and safety in unresectable HCC with a liver function Child–Pugh grade A or B [32]. Before and after the propensity-matched analysis of the retrospective cohort of 222 patients, the combination group was found to have a higher ORR (before PSM—88.9% vs. 30.5%, *p* < 0.001; after PSM—88.6% vs. 28.6%, *p* < 0.001) and DCR (before PSM—95.9% vs. 69.5%, *p* < 0.001; after PSM—94.3% vs. 72.9%, *p* < 0.001). The median PFS and OS were also significantly better in the combination group compared to the TACE group after PSM (PFS: 12.7 vs. 6.1 months, *p* = 0.005; OS: 19.4 vs. 13.0 months, *p* = 0.023). Wu et al. performed a single-arm prospective study on unresectable HCC with China liver cancer staging 2–3 and liver function Child–Pugh class A, utilizing the same combination of TACE plus lenvatinib plus camrelizumab, and found that the ORR was 76.4% and the DCR was 85.5% for the combination [33]. Importantly, 54.5% of the patients were converted to resectable HCC, and 52.7% of the patients underwent a resection. Zhang et al. performed a retrospective study on similar lines in unresectable HCC, but compared the combination of lenvatinib/sorafenib plus immune checkpoint inhibitors (ICIs) (camrelizumab, sintilimab, and atezolizumab) with or without TACE in a total of 215 patients [34]. After the inverse probability weighting, the ORR was significantly higher in the TACE-ICI-TKI group compared to the TKI-ICI group (50.9% vs. 28.4%, *p* < 0.001). The median PFS and OS were significantly longer in the TACE-TKI-ICI group than in the TKI-ICI group (PFS: 9.1 vs. 5.0 months, *p* = 0.005; OS: 19.1 vs. 12.7 months, *p* = 0.002). Treatment-related grade 3 or 4 adverse events were similar between the two groups (*p* = 0.437).

Lenvatinib and a programmed cell death protein 1 (PD-1) inhibitor combined with TACE has shown great promise in patients with initially unresectable HCC for a conversion surgery as compared to initial surgery. From before and after the propensity-matched analysis, it was discovered that the conversion surgery group could have a significantly improved event-free survival (before PSM—*p* < 0.001; after PSM—*p* = 0.001). The conversion surgery group also had a significantly lower incidence of microvascular invasion compared to the initial surgery group (3.1 vs. 50.4%, *p* < 0.001) [35]. Wang et al. conducted a meta-analysis in 2024 involving 13 cohort studies comprising 1279 patients. The studies combined TACE, lenvatinib, and PD-1 inhibitors, which was found to significantly improve the OS (HR—0.50; *p* < 0.001), PFS (HR—0.48; *p* < 0.001), ORR (1.75; *p* < 0.001), and DCR (RR1.41; *p* < 0.001) compared with other treatment regimens. The AE profile did not differ considerably between the groups [36].

Li et al. performed a meta-analysis of 16 studies comprising 1650 cases of unresectable HCC to assess the value of lenvatinib-based regimens for the conversion therapy of unresectable HCC [37]. The regimens assessed included lenvatinib alone; lenvatinib plus ICIs; lenvatinib plus locoregional therapy; and triple therapy with lenvatinib, ICIs, and locoregional therapy. The pooled conversion rate was found to be especially high for the regimens involving lenvatinib plus ICIs, with (35%) or without (23%) locoregional therapy. The pooled ORR was 49% for the lenvatinib plus ICI group and 69% for the lenvatinib, ICI, and locoregional therapy group. Zhang et al. conducted a retrospective study involving 108 patients between 2019 and 2022 to compare the efficacy of adding TACE to the combination of ICIs and TKIs (lenvatinib or sorafenib) in unresectable HCC; they found that the ORR and the DCR were significantly higher in the TACE-TKI-ICI group (ORR: 63.0 vs. 29.6%, *p* < 0.001; DCR: 85.2% vs. 53.7%, *p* < 0.001) [38]. The median PFS and OS were also significantly better in the TACE-ICI-TKI group (PFS: 9.9 vs. 5.8 months, *p* = 0.026; OS: not reached vs. 18.5 months, *p* = 0.003). Table 1 summarizes the findings of all the retrospective studies evaluating the combination of TACE and lenvatinib in HCC.

**Table 1:** Studies evaluating the combination of TACE and lenvatinib in HCC.

Author	Year	Country	Superior Group	Comparison Group	ORR	PFS	OS
Kawamura et al. [23]	2020	Japan	Lenvatinib + TACE	Lenvatinib	The ORR was significantly higher for the heterogeneous enhancement pattern (85 vs. 53%, respectively) (*p* = 0.028).	Not assessed.	Lenvatinib–TACE sequential therapy was associated with significant PPS (HR—0.08; *p* = 0.023).
Fu et al. [22]	2021	China	TACE + lenvatinib	TACE alone	ORR in the TACE + lenvatinib group was higher than that in the TACE group (68.3% vs. 31.7%, *p* < 0.001).	PFS was improved for patients in the TACE + lenvatinib group more than those in the TACE group (1 y PFS rate: 78.4% vs. 64.7%; 2 y PFS rate: 45.5% vs. 38.0%; *p* < 0.001; HR = 0.343; 95% CI: 0.198–0.595)	The 1 y and 2 y OS rates were improved for TACE + lenvatinib vs. the TACE group (88.4% and 79.8% vs. 79.2% and 49.2%) (*p* = 0.047; hazard ratio [HR] = 0.466; 95% CI = 0.226–0.886).
Ando et al. [24]	2021	Japan	TACE + lenvatinib	Lenvatinib alone	The ORR was similar for the TACE + lenvatinib and the lenvatinib groups (63.2%).	Median PFS was improved for the TACE + lenvatinib group vs. the lenvatinib-alone group (11.6 vs. 10.1 months).	The OS of the TACE + lenvatinib group was significantly higher than that of the lenvatinib group (median survival time: not reached vs. 16.9 months; *p* = 0.007)
Tada et al. [25]	2021	Japan	Early lenvatinib	No lenvatinib	Not assessed.	Not assessed.	Lenvatinib therapy was significantly associated with overall survival in patients with HCC beyond the up-to-7 criteria (HR, 0.230; 95% CI, 0.059–0.904; *p* = 0.035).
Shimose et al. [26]	2021	Japan	Lenvatinib + TACE	Lenvatinib	Not assessed.	Not assessed.	The overall survival of the TACE group was significantly higher than that of the non-TACE group (median OS: not reached vs. 16.3 months; *p* = 0.01).
Yang et al. [31]	2021	China	TACE + lenvatinib	TACE + sorafenib	Before and after PSM, ORR in the TACE–lenvatinib group was significantly better than that in the TACE–sorafenib group (before PSM: 60.7% vs. 38.9% (OR 2.43; 95% CI: 1.13–5.23; *p* = 0.022); after PSM: 66.8% vs. 33.3% (OR 0.85; 1.05–6.90; *p* = 0.037)).	After PSM, the median PFS in the TACE–lenvatinib group was significantly better than that in the TACE–sorafenib group (10.6 vs. 5.4 months; HR: 2.62; 95% CI: 1.43–4.80; *p* = 0.002).	The median OS after PSM in the TACE–lenvatinib group was significantly better than that for the TACE–sorafenib group (18.97 months vs. 10.77 months; HR 2.21; 95% CI: 1.12–4.38; *p* = 0.022)
Chen et al. [27]	2022	China	TACE + lenvatinib in portal vein tumor thrombosis	Indirect comparison with lenvatinib in the REFLECT trial	The ORR and DCR were 75% and 91.7%, respectively, for the TACE–lenvatinib group.	The median PFS for the TACE–lenvatinib group was 6.15 months.	The median OS was 16.9 months for the TACE + lenvatinib group.
Tang et al. [32]	2024	China	TACE + lenvatinib + camrelizumab	TACE	Before and after PSM, the ORR was higher in the TACE + lenvatinib + camrelizumab group than in the TACE group (before PSM: 88.9% vs. 30.5%, *p* < 0.001; after PSM: 88.6% vs. 28.6%, *p* < 0.001).	After PSM, the median PFS was longer in the TACE + lenvatinib + camrelizumab group than in the TACE group (12.7 vs. 6.1 months, *p* = 0.005).	The median OS was longer in the TACE + lenvatinib + camrelizumab group than in the TACE group (19.4 vs. 13.0 months, *p* = 0.023).
Zhang et al. [34]	2023	China	TACE + ICI + TKI	ICI + TKI	After PSM, the ORR was higher in the TACE-TKI-ICI group (50.9% vs. 28.4%, *p* < 0.001).	After PSM, the median PFS was significantly longer in the TACE-TKI-ICI group than in the TKI-ICI group (PFS: 9.1 vs. 5.0 months; *p* = 0.005).	After PSM, the median OS was significantly longer in the TACE-TKI-ICI group than in the TKI-ICI group (OS: 19.1 vs. 12.7 months; *p* = 0.002).
Zhang et al. [38]	2024	China	TACE + ICI + TKI	ICI + TKI	After PSM, the ORR and DCR were higher in the TACE-TKI-ICI group (ORR: 63.0% vs. 29.6%, *p* < 0.001; DCR: 85.2% vs. 53.7%, *p* < 0.001).	After PSM, the median PFS was significantly longer in the TACE-TKI-ICI group (9.9 vs. 5.8 months; *p* = 0.026).	After PSM, the median OS was significantly improved in the TACE-TKI-ICI group (not reached vs. 18.5 months; *p* = 0.003).
Lin et al. [29]	2024	Taiwan	Locoregional therapy + lenvatinib	Lenvatinib	Not assessed	Not assessed.	Patients who received subsequent locoregional therapy had significantly better survival than those who did not receive it (1st-year cumulative survival rate: 70% vs. 27%; log-rank *p* = 0.003).
Wang et al. [30]	2023	China	Lenvatinib + TACE + pembrolizumab	Lenvatinib + TACE	The ORR of the combined group and the lenvatinib-TACE group were 44.4% and 20% (*p* = 0.059) according to the mRECIST criteria.	The median PFS was longer in the combined group compared to the lenvatinib–TACE group (11.7 months vs. 8.5 months, * p * = 0.028 ).	Patients with unresectable HCC who received combined therapy had a longer OS than those who underwent lenvatinib–TACE therapy (26.8 months vs. 14.0 months; *p* = 0.027).
Wu et al. [33]	2024	China	Lenvatinib + TACE + camrelizumab		The ORR was 76.4% and the DCR was 85.5% per modified RECIST.	The median PFS was not reached.	The median OS was not reached.

Abbreviations: TACE: transarterial chemoembolization; PPS: post-progression survival; HR: hazard ratio; OS: overall survival; PFS: progression-free survival; OR: odds ratio; ORR: overall response rate; DCR: disease control rate; ICI: immune checkpoint inhibitor; TKI: tyrosine kinase inhibitor.

#### 3.1.2. Clinical Trials Exploring Lenvatinib and TACE

There have not been as many clinical trials as retrospective studies evaluating the TACE plus lenvatinib combination. Ding et al., in 2021, conducted an important randomized trial evaluating this combination, with sorafenib and TACE as the first-line treatment for HCC with PVTT [39]. They enrolled 64 patients who were randomized to either arm and found that, after 16.1 months of follow-up, the patients in the lenvatinib arm had a higher median time-to-progression (TTP) and ORR compared to the sorafenib arm (TTP: 4.7 vs. 3.1 months; HR—0.55, *p* = 0.029; ORR: 53.1% vs. 25.0%; *p* = 0.039). In 2023, Peng et al. published the results of the LAUNCH trial, a phase 3 RCT comparing the clinical outcomes of lenvatinib combined with TACE versus lenvatinib monotherapy in patients with advanced HCC [40]. The study subjects (*n* = 338) were randomized 1:1 to either group at 12 centers in China. After a median follow-up of 17 months, the OS was significantly longer in the lenvatinib–TACE group compared to the lenvatinib monotherapy group (17.8 vs. 11.5 months; *p* < 0.001). The median PFS was also improved in the lenvatinib–TACE group (10.6 vs. 6.4 months; *p* < 0.001). The response rate using the mRECIST criteria was 54.1% in the lenvatinib–TACE group compared to 25% in the lenvatinib monotherapy group. From a subgroup analysis, there was no significant difference in the outcomes between the use of conventional TACE vs. drug-eluting bead TACE (DEB-TACE). The most significant grade 3–4 AEs in the lenvatinib–TACE group included elevated liver enzymes and hyperbilirubinemia. From the subgroup analyses, the most favorable outcomes, in terms of survival, were especially noted among those with a significant tumor burden, as evidenced by the presence of PVTT, AFP levels ≥ 400 ng/mL, ≥3 intrahepatic lesions, and a main tumor size ≥ 5 cm. On the flipside, the patients in the lenvatinib–TACE group required a longer duration of treatment compared to those on lenvatinib monotherapy (8.2 vs. 5.1 months).

### 3.2. Lenvatinib with Other Locoregional Therapies

Although not as many studies have included the use of TARE in an HCC setting with lenvatinib, theoretically it should provide the same benefit as TACE. Table 2 outlines the studies that have been conducted evaluating the combination of TKI with TARE. Ocal et al., in 2022, analyzed the follow-up images of 177 patients (104 in the sorafenib arm versus 73 patients in the combination arm) and found that the combination arm had a significantly higher ORR (61.6% vs. 29.8%; *p* < 0.001) and a higher complete response rate (13.7% vs. 3.8%; *p* = 0.022) [41]. The PFS (8.9 vs. 5.4 months; *p* = 0.022) and hepatic PFS (9.0 vs. 5.7 months; *p* = 0.014) were also significantly better in the combination arm. The enhanced response, however, did not translate into prolonged survival. Another combination study using Yttrium90 (Y90) along with sorafenib or nivolumab was conducted by Chung et al. in patients with HCC with hepatic vein or inferior vena cava invasion. They found that the patients receiving combination therapy with radioembolization and systemic agents saw an increased OS of 20.6 months and a median PFS of 8.2 months [42]. This report also included two important retrospective studies conducted by Cooke et al. and Spreafico, establishing the utility of radioembolization in an HCC setting [43,44].

Lenvatinib has also been explored in combination with radiofrequency ablation (RFA) for intermediate-stage HCC. Wang et al. compared patients receiving lenvatinib monotherapy with patients receiving lenvatinib initially followed by subsequent RFA. The combination demonstrated a higher ORR compared to the monotherapy group (100% vs. 76.9%). The PFS and OS were also higher in the combination group (12.5 vs. 5.5 months; 21.3 months vs. 17.1 months). From their Cox regression analysis, the treatment strategy was found to be an independent factor for PFS, while no statistically significant differences were observed with respect to the AEs, except for liver enzyme elevation (*p* = 0.007) in the combination group [45]. Wang et al. assessed the combination of lenvatinib plus sintilimab with or without the presence of RFA and found that the combination of lenvatinib plus sintilimab plus RFA showed a significantly higher ORR, OS, and PFS compared to lenvatinib plus sintilimab alone (ORR: 40.0% vs. 20.9%, *p* = 0.022; PFS: 12 months vs. 8 months, *p* = 0.013; OS: 24 vs. 18 months, *p* = 0.037) [46].

TACE has also been studied in combination with microwave ablation (MWA) with or without lenvatinib in HCC by Men et al. In a recently published retrospective study of 67 patients with HCC, they found that, while the PFS was not reached for the group treated with TACE plus MWA plus lenvatinib, the PFS was 17.05 months if only treated with TACE and MWA. From the regression analysis, the failure to combine treatment with an adjuvant therapy was also identified as an independent risk factor for tumor recurrence [47].

**Table 2:** Studies evaluating the combination of other locoregional modalities with tyrosine kinase inhibitors in HCC.

Author	Year	Country	Superior Group	Comparison Group	ORR	PFS	OS
Men et al. [47]	2024	China	TACE+ MWA+ lenvatinib	TACE+ MWA	Not assessed.	The median PFS at 1 year was significantly better in the treatment arm compared to the control arm (not reached vs. 17.05 months, *p* = 0.035).	Not assessed.
Ocal et al. [41]	2022	European Union	TARE+ sorafenib	Sorafenib	The combination arm had significantly higher ORR (61.6% vs. 29.8%, *p* < 0.001).	PFS (median 8.9 vs. 5.4 months, *p* = 0.022) and hepatic PFS were significantly better in the combination arm (9.0 vs. 5.7 months, *p* = 0.014).	There was no difference in overall survival between study arms (HR, 1 [0.76–1.5]; *p* = 0.77).
Wang et al. [45]	2022	Japan	Lenvatinib + RFA	Lenvatinib	The combination group exhibited a higher ORR (100%) than the monotherapy group (76.9%).	The combination group had a longer PFS (12.5 months) than the monotherapy group (5.5 months).	The median OS was 21.3 months for the combination group and 17.1 months for the monotherapy group.
Wang et al. [46]	2024	China	Lenvatinib + sintilimab + RFA (R-L-S)	Lenvatinib + sintilimab (L-S)	The R-L-S group had a significantly higher ORR than L-S group (40.0% vs. 20.9%; *p* = 0.022).	Patients in the R-L-S group had improved median PFS (12 vs. 8 months; *p* = 0.013) compared to L-S alone.	Patients in the R-L-S group had improved median OS (24 vs. 18 months; *p* = 0.037) compared to L-S alone.

Abbreviations: TACE: transarterial chemoembolization; MWA: microwave ablation; PFS: progression-free survival; ORR: objective response rate; TARE: transarterial radioembolization; OS: overall survival.

## 4. Future of Combination Therapies and Clinical Trials in Progress

The success of combination therapies with sorafenib and lenvatinib has paved the way for other combination therapies in locoregional HCC. The triple combination of lenvatinib, the PD-1 inhibitor sintilimab, and TACE has been explored in a prospective study involving 116 patients with HCC and portal vein thrombosis [48]. The median PFS and the median OS were significantly longer in the TACE–lenvatinib–sintilimab group compared to the TACE–lenvatinib group (PFS: 4.17 vs. 3.33 months, *p* = 0.01; OS: not reached vs. 8.53 months, *p* = 0.003). The ORR and DCR, although not reaching statistical significance, were also slightly higher in the triplet group than in the doublet group. Another popular combination that is being researched includes TACE plus atezolizumab and bevacizumab for intermediate-stage HCC [49]. In a multi-center retrospective study including 21 patients, the best ORR and DCR according to the mRECIST criteria were 61.9% and 100%, respectively. Another recently published accomplishment in the field of combination therapy for HCC was achieved by the EMERALD-1 trial, which combined TACE with durvalumab with or without bevacizumab in unresectable HCC patients eligible for embolization. With a total of 616 patients, the PFS was significantly improved for durvalumab plus bevacizumab plus TACE compared to TACE alone (15 months vs. 8.2 months, *p* = 0.032) [50]. An offshoot of the EMERALD-1 trial, which may change the existing treatment paradigm, is the EMERALD-3 trial, which will evaluate the combination of TACE with tremelimumab, durvalumab, and lenvatinib in intermediate-stage HCC. The primary endpoints for this study will include the PFS with other additional endpoints, including the health-related quality of life and safety [51]. Another study to watch is the LEAP-012 trial, comparing TACE plus lenvatinib plus pembrolizumab compared to TACE alone. The primary endpoints for this study will be the OS and PFS, and it has gained in importance after the recent release of the results of the LEAP-002 trial [52]. CheckMate 74W is another relevant study currently underway that aims to assess the efficacy of the combination of nivolumab plus ipilimumab plus TACE in intermediate-stage HCC [53]. A complete list of the ongoing phase 3 clinical trials in intermediate-stage HCC using a combination of systemic agents with locoregional therapy appears in Table 3.

## 5. Conclusions

Lenvatinib is an important part of the arsenal in the treatment of HCC. With the development of new therapeutics in the advancing HCC field, lenvatinib has found a new role in combination therapies with locoregional treatments for better tumor control of intermediate-stage HCC. Despite the advent of ICIs, lenvatinib retains its role of improving the outcomes of advanced HCC. On the other hand, the combination of lenvatinib and TACE has found a role in the management of intermediate-stage HCC (BCLC B) and HCC with portal vein tumor thrombosis. Combining Y90 with lenvatinib has also been found to have better outcomes, particularly in HCC with hepatic/portal vein invasion. This role needs to be explored further with the help of well-designed clinical trials and studies to elucidate its clear superiority and guidelines for use. It is also an important case study for not abandoning older therapies with the development of newer therapeutics, but establishing new roles and indications for them.

The HCC treatment landscape has undergone a paradigm shift in the last decade. This success story can be attributed to the implementation of immunotherapy and new ways to utilize combination therapies. The ongoing research holds huge promise, and the future looks extremely bright for the use of combination therapies for curative intent in intermediate-stage HCC.

## Figures and Tables

**Table 3:** Table showing upcoming trials using locoregional therapy with systemic agents.

Trial Name	Comparison Group	Sample Size	Study Period	Primary Endpoint	Secondary Endpoint
ChiCTR2200066830	TACE + lenvatinib +/− sintilimab	218	1 January 2023	TTP	OS, ORR, safety
ABC-HCC trial	Atezolizumab + bevacizumab vs. TACE for intermediate HCC	434	7 June 2021 to July 2027	Time to failure of treatment strategy	OS, ORR, TTP, PFS, DOR, duration of treatment, QoL, toxicity, time to deterioration of liver function, time to loss of systemic treatment options
REPLACEMENT study	Atezolizumab + bevacizumab for TACE-unsuitable patients	70	13 November 2020–end date not provided	PFS	PFS, ORR, duration of response (DOR) per mRECIST, PFS, ORR, DOR per RECIST ver.1.1, OS, safety
EMERALD-1	TACE + durvalumab +/− bevacizumab	724	30 November 2018 to 31 August 2026	PFS	PFS, OS, QoL, HrQoL
EMERALD-3	TACE + tremelimumab + durvalumab + lenvatinib vs. TACE + tremelimumab + durvalumab	760	28 March 2022 to 26 February 2027	PFS	PFS, OS
LEAP-012	TACE + lenvatinib + pembrolizumab vs. TACE	450	20 May 2020 to 31 December 2029	PFS, OS	PFS, ORR, DCR, DOR, TTP, toxicity/adverse events
CheckMate 74W	Nivolumab + ipilimumab + TACE in intermediate HCC	26	14 September 2020 to 12 December 2023	Toxicity	
PETAL1	Pembrolizumab following TACE	26	28 January 2018 to 23 March 2023	Safety and tolerability	PFS
Pro2021001725	Tislelizumab following TACE	35	25 July 2022 to 1 June 2027	PFS	Time to metastatic disease, OS, ORR, DCR, DOR, safety profile, biomarker response
NCT03937830	Durvalumab + bevacizumab + tremelimumab +TACE	27	10 March 2021 to 31 December 2025	PFS	Best overall response (BOR), OS, safety
